# A comparison study of radiation effective dose in ECG-Gated Coronary CT Angiography and calcium scoring examinations performed with a dual-source CT scanner

**DOI:** 10.1038/s41598-019-40758-5

**Published:** 2019-03-13

**Authors:** Akmal Sabarudin, Tiong Wei Siong, Ang Wee Chin, Ng Kwan Hoong, Muhammad Khalis Abdul Karim

**Affiliations:** 1 Diagnostic Imaging & Radiotherapy Program, Faculty of Health Sciences, Universiti Kebangsaan Malaysia, 50300 Kuala Lumpur, Malaysia; 2 Department of Physics, Faculty of Science, Universiti Teknologi Malaysia, 81300 Johor Bharu, Johor Malaysia; 3 Department of Biomedical Imaging, Universiti of Malaya Medical Centre, 50603 Kuala Lumpur, Malaysia; 4 Department of Physics, Faculty of Science, Universiti Putra Malaysia, 43400 Serdang, Selangor Malaysia; 5 Center for Diagnostic Nuclear Imaging, Faculty of Medicine Universiti Putra Malaysia, 43400 Serdang, Selangor Malaysia

## Abstract

In this report we have evaluated radiation effective dose received by patients during ECG-gated CCTA examinations based on gender, heart rate, tube voltage protocol and body mass index (BMI). A total of 1,824 patients were retrospectively recruited (1,139 men and 685 women) and they were divided into Group 1 (CCTA with calcium scoring), Group 2 (CCTA without calcium scoring) and Group 3 (only calcium scoring), where the association between gender, heart rate, tube voltage protocol and body mass index (BMI) were analysed. Examinations were performed using a retrospective ECG-gated CCTA protocol and the effective doses were calculated from the dose length product with a conversion coefficient of 0.026 mSv.mGy^−1^cm^−1^. No significant differences were observed in the mean effective dose between gender in all groups. The mean estimated dose was significantly higher when the heart rate was lower in Group 1 (*p* < 0.001) and Group 2 (*p* = 0.002). There were also significant differences between the mean effective dose in tube voltage protocol and BMI among the three groups. The mean effective dose was positively correlated with BMI (*p* < 0.001), but inversely related to the heart rate. This study supported the theory that a high heart rate, low tube voltage and low BMI could significantly reduce radiation dose exposure.

## Introduction

Coronary computed tomography angiography (CCTA) is a non-invasive diagnostic tool for detecting coronary heart disease that has low radiation exposure on patients compared with conventional angiography^1,2^. Despite its benefits, the radiation dose is still a concern among clinicians and CT scan manufacturers, and numerous techniques have been introduced to reduce its side effects on patients and medical personnel^2^.

Prospective ECG-gated CCTA has been shown to be the most efficient alternative in minimising radiation exposure while maintaining image quality compared with retrospective ECG-gated CCTA. The effective radiation dose exposure in prospective ECG-gated protocols have been proven to be between 5 and 7 times lower compared with retrospective ECG-gated CCTA. A phantom study has found that the effective dose in retrospective protocol could be as high as 18.2 ± 8.3 mSv in dual-source CT (DSCT) scans and 28.3 ± 7.0 mSv in single-source CT (SSCT) scans^3^. Although prospective ECG-gating CCTA has low effective radiation dose, its use is severely restricted for cardiac patients with high and fluctuating heart rates.

The optimal heart rate for the procedure is between 65 and 75 beats per minute (bpm) because that range has the best diastolic phase to capture images of coronary arteries with minimal artifacts^4–6^. Currently, functional analysis of the heart can be performed using prospectively ECG-triggered sequential single cardiac phase CT images obtained through CCTA — derived from the fractional flow reserve (FFR_CT_)^7^ or by using myocardium analysis^8^. In contrast, retrospective ECG-gated CCTA is able to provide reconstruction of motion-free images with greater flexibility, thus allowing coronary arteries to be viewed at any R-R interval^9^.

To improve the CCTA image of patients with high heart rate, beta-blockers (metoprolol) or calcium channel blockers (Diltiazem and Verapamil) can be prescribed^10^. But these drugs are contraindicated in patients with a history of significantly-impaired left ventricle function and heart failure because they reduce myocardial contractility. Currently, Ivabradine is the only drug that has pure negative chronotropic effects in inhibiting the “I” (funny) ion channels of sinoatrial nodes to lower the heart’s natural pace-making activity without binding to β-adrenergic receptors. Thus, Ivabradine provides a safer option to reduce the patients’ heart rate^11^. These dedicated medicines can be used to lower the heart rate for patients undergoing prospective ECG-gated CCTA procedure.

Sometimes, radiographers choose to use unoptimized CT protocols and may end up mis-centering the patients’ position, leading to increased effective radiation dose exposure. For whatever reason, it is important to apply the principle of ALARA (As Low as Reasonably Achievable) in all radiological procedures. There are several protocols that can be used cardiac CT inclusive contrast (CCTA) and non-contrast (calcium scoring) studies^12,13^. Both protocols are being implemented in clinical practice regardless of patient condition. However, the accumulated dose for both procedures can be high and it should be taken into consideration if patient safety is to be given priority. The implementation of either a single protocol or both remains debatable^14^.

Therefore, the purpose of this study is to evaluate the effective radiation doses received by patients who had undergone retrospective ECG-gated CCTA and calcium coronary CT scan, and compare them according to gender, heart rate, BMI and tube voltage protocol.

## Materials and Methods

### Study subjects

The research protocol was approved by the ethic committee of Institut Jantung Negara (IJN) which waived patient consent form for the retrospective analysis with an approval ID: IJNEC/05/2013(01). A total of 1,824 patients comprising 1,139 men and 685 women (aged 23 to 88 with a mean of 56.56 ± 10.65 years) who underwent CCTA between February 2015 and January 2016 in IJN were retrospectively recruited. Paediatric patients (below age 18) and multiple scan area cases were excluded.

Sublingual nitro-glycerine was administered when the patient was lying down, and beta-blockers were given only if their heart rate was more than 85 bpm. Tube voltage protocol was adjusted based on patients’ height and weight. Patient information (height, weight, heart rate, blood pressure) were noted and the body mass index (BMI) was calculated.

In this study, patients were divided into three groups, namely Group 1 (CCTA with calcium scoring), Group 2 (CCTA without calcium scoring) and Group 3 (calcium scoring only). Group 1 consists of first-time patients presenting with major risks for coronary artery disease and myocardial infarction, such as high blood cholesterol, family history of heart attack, diabetes, high blood pressure, smoking, overweight or obese, and physical inactivity. Group 2 consists of patients with a history of heart attack and had undergone cardiac interventions like coronary artery bypass graft (CABG), post-stenting and pacemaker implantation. Group 3 represents patients with severe/major calcifications in coronary arteries, which did not require further CCTA and likely considered for screening purposes only.

### CT acquisition parameters

CT examinations were performed using the 64-slice dual-source Siemens Somatom Definition Flash scanner (Siemens Medical Solution, Erlangen, Germany) according to parameters in Table 1. Tube voltage protocol was selected based on the patients’ BMI as indicated in Table 2. These parameters were standard protocol in IJN, and details like effective mAs and scanning time were tabulated in Table 3. Patients were positioned supine at the centre of the scanner gantry and a coronal topogram was obtained.

**Table 1:** Standard acquisition parameters for coronary calcium scoring and CCTA.

Acquisition parameter	Calcium scoring	CCTA
kV	100–140	100–140
mA	30–250	300–500
Effective mAs	80	350
Slice collimation	2 × 64 × 0.6 mm with Z-flying focal spot*	2 × 64 × 0.6 mm with Z-flying focal spot*
Slice thickness	3.0 mm	0.6 mm
Pitch factor	1.0	0.2–0.43

*Actual nominal beam width was 2 × 32 × 0.6 mm.

**Table 2:** Selection of tube voltage (kV) based on BMI.

Group	Category BMI	kV selection	Number of patients
1	Underweight	100 or 120 kV	29
	(BMI < 18.5 kg/m²);		
2	Normal weight	100 or 120 kV	547
	(18.5–24.9 kg/m²);		
3	Pre-obesity	100 or 120 kV	778
	(25–29.9 kg/m²)		
4	Obesity class I	120 or 140 kV	344
	(30–34.9 kg/m²)		
5	Obesity class II	120 or 140 kV	88
	(35–39.9 kg/m²);		
6	Obesity class III	140 or160 kV	38
	(BMI ≥ 40 kg/m²)

**Table 3:** Differences in mean estimated effective dose (mSv) between gender and heart rate.

Group	Male	Female	*p* value	≤65 bpm	>65 bpm	*p* value
1	19.05 ± 8.74	19.75 ± 9.49	0.245	20.38 ± 9.64	18.81 ± 9.41	0.000
2	25.65 ± 12.93	23.76 ± 12.93	0.149	27.53 ± 13.23	23.37 ± 12.30	0.002
3	4.18 ± 3.03	5.10 ± 3.33	0.056	4.44 ± 1.97	4.26 ± 2.54	0.160

Based on the program, CT coronary calcium scoring and CCTA were performed, with the scanning range between the ascending aorta and heart apex to include the entire cardiac structure. In the CCTA scanning protocol, ECG-pulsing was used to steer the scan acquisition according to heart rhythm and to reduce radiation dose. ECG-pulsing window was set between 30% and 80% of the R–R interval (least motion for the coronary arteries) with a pitch ranging from 0.2 to 0.43, which was automatically adapted to the heart rate. For CT coronary calcium scoring, the ECG-pulsing window was set between 60% and 75% of the R-R interval with a pitch of 1.0. The ECG-pulsing kept the tube current at the highest level for user-defined R-R interval and used a lower tube current (20% reduction) for the remaining phase that eventually led to low radiation dose exposure.

The test bolus technique was used to calculate the scan-time delay with peak contrast enhancement. Patients were injected intravenously with 20 ml of low osmolar iodinated contrast media (Iomeron 350, Bracco, UK) and trail scans were performed at the ascending aorta to monitor contrast enhancement. Time to peak enhancement was determined through the contrast enhancement curve. Thus, the scan-time delay was calculated by adding the time to peak enhancement and acquisition trigger time. Another 60 mL of contrast medium was injected into patients using a power injector at a flow rate of 5.0–6.0 mL/s, followed by 60 ml of saline flushing at the same flow rate.

The CCTA scan for patients with percutaneous cardiac intervention (PCI) and CABG was made cranio-caudally with bigger scan range between the arch of aorta and the apex of the heart to include the entire heart and the ligation of the grafts. The patients were instructed to hold their breath during the scan acquisition. Automatic tube current modulation and automatic ECG-pulsing were used to further reduce radiation exposure.

### Effective dose estimation

The dose length product (DLP) was obtained retrospectively from records of the daily patients’ list. The effective dose of CCTA was estimated from the DLP product using a conversion coefficient value (*k*) of 0.026 mSvmGy^−1^cm^−1^ as reported by Huda *et al*. for coronary CT examinations^15^. The estimated effective dose (E) is calculated below:

$${\rm{E}}={k}\times {\rm{DLP}}$$

### Statistical analysis

Data were analyzed using IBM SPSS version 22.0 (IBM Corporation, Armonk, New York, USA). The Kolmogorov-Smirnov Test was used to determine the normality of the estimated effective dose. The quantitative variables were expressed as a mean ± standard deviation. Differences between the two groups were determined using the Mann-Whitney U Test (*p* < *0.05*), while the Kruskal-Wallis H Test was used for more than two groups (*p* < 0.05). The mean comparisons between variables were presented descriptively. Spearman’s correlation analysis was used to determine the association between effective dose and heart rate, as well as BMI.

## Results

### Patient characteristics and scan parameters

**Figure 1:**
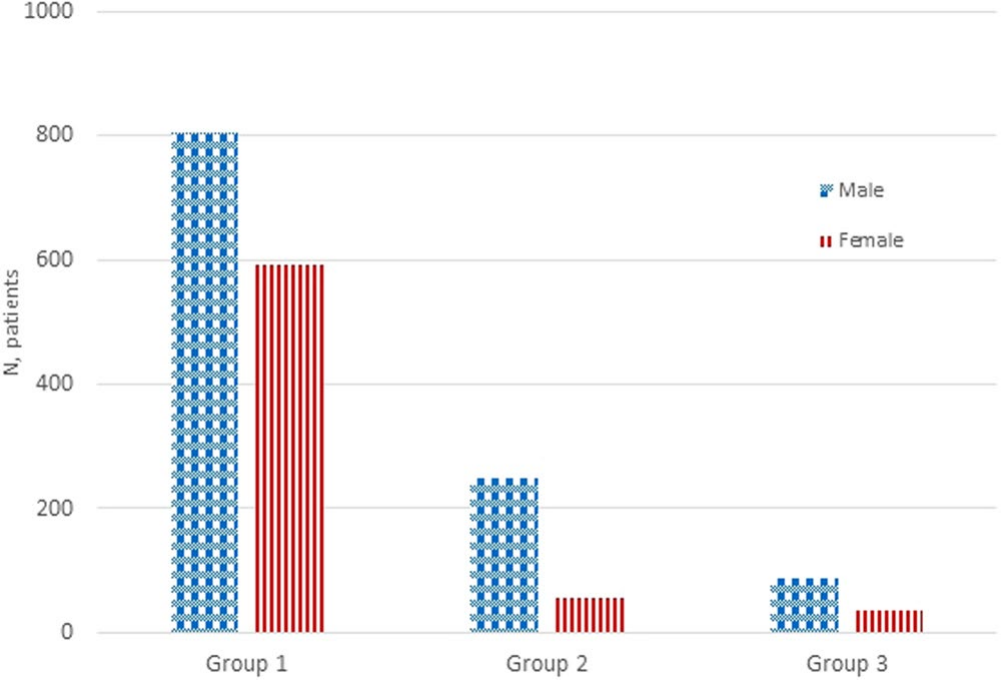
Distribution of gender in each examination group. The groups are divided into Group 1: CCTA with calcium score, Group 2: CCTA only, and Group 3: calcium score only.

**Figure 2:**
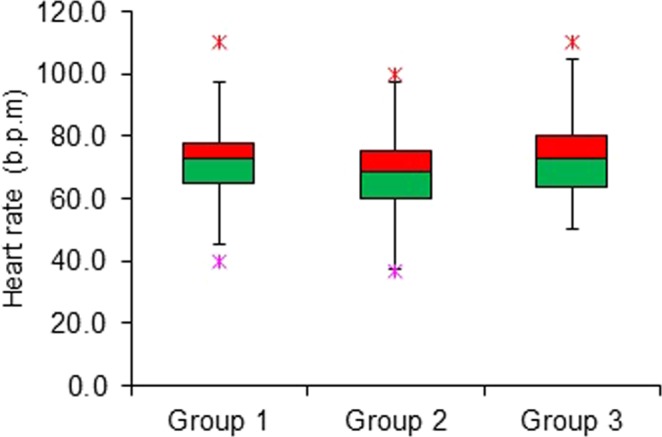
Boxplots indicating the heart rate of patients in each group.

The patients were characterized by gender and examination groups as shown in Fig. 1. The boxplots in Fig. 2 show the heart rate of patients, where 1,197 (65.65%) of them had more than 65 bpm., and 627 (34.35%) had less or equal to 65 bpm. The mean heart rate was 71 ± 11 bpm (range 37–123 bpm) and mean BMI was 27.54 ± 5.04 (range 13.62–61.14).

### Radiation dose comparison

**Figure 3:**
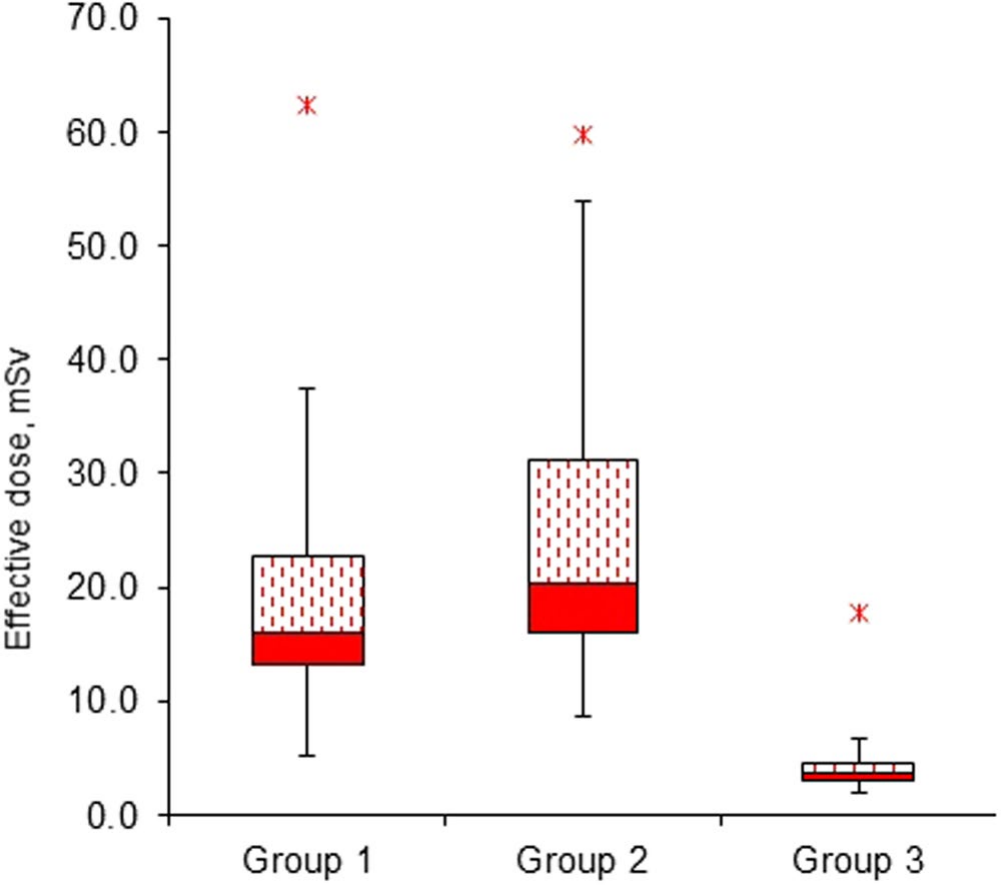
Effective dose received by each group.

In radiation dose analysis, Fig. 3 presents the estimated effective dose received by different groups. It was apparent that Group 2 patients had the highest median value and Group 3 patients received the lowest dose. In Table 3, the Mann-Whitney U test proved that there was no difference between the mean effective dose and gender in all examination groups. However, the estimated effective doses were significantly different between heart rate in Group 1 (*p* < 0.000) and Group 2 (*p* = *0.002*) patients, but not Group 3. Interestingly, the doses were higher when the patients’ heart rate was low.

Table 4 shows the comparison between the estimated effective dose and tube voltage protocols using the Kruskal-Wallis H-test. The effective doses in different voltage protocols were significantly different in all groups. An interesting observation was the mean effective dose in Group 2 using a tube voltage of 120 kV, which was the highest value compared with all groups. As indicated in Table 5, the estimated effective dose among the BMI category was also found to be statistically significant among all groups.

**Table 4:** The mean estimated effective dose (mSv) and voltage protocols among groups.

Group	100 kV	120 kV	140 kV	160 kV	*p*-value
1	14.87 ± 4.36	27.96 ± 9.53	34.75 ± 4.01	11.08 ± 0.00	0.000
2	18.78 ± 7.28	37.39 ± 12.49	28.11 ± 0.00	N.A.	0.000
3	3.89 ± 2.38	4.72 ± 3.57	6.55 ± 0.00	N.A.	0.031

**Table 5:** The mean estimated effective dose (mSv) and BMI categories among groups.

BMI categories	1	2	3	4	5	6	*p*-value
Group 1	13.40 ± 2.20	14.86 ± 5.44	17.93 ± 8.84	27.06 ± 10.13	28.97 ± 7.82	31.63 ± 10.45	0.000
Group 2	23.99 ± 15.37	19.36 ± 12.41	24.54 ± 11.68	32.83 ± 12.50	34.04 ± 7.29	32.17 ± 15.25	0.000
Group 3	2.67 ± 0.50	3.16 ± 0.88	4.05 ± 2.08	5.30 ± 2.24	5.75 ± 2.09	11.90 ± 8.37	0.000

1 = Underweight (BMI < 18.5 kg/m²); 2 = Normal weight (BMI 18.5–24.9 kg/m²); 3 = Pre-obesity (BMI 25–29.9 kg/m²); 4 = Obesity class I (BMI 30–34.9 kg/m²); 5 = Obesity class II (BMI 35–39.9 kg/m²); 6 = Obesity class III (BMI ≥ 40 kg/m²).

**Figure 4:**
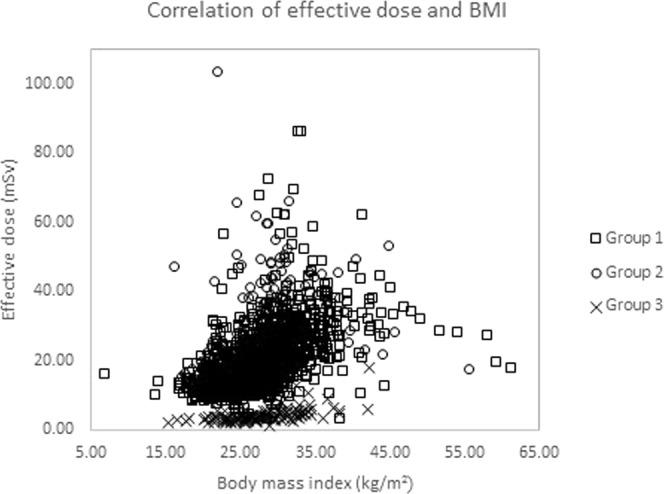
The graph shows correlation analysis of the estimated effective dose depending on BMI. Positive correlation was shown in all three groups (Group 1, r = 0.610; Group 2, r = 0.516; and, Group 3, r = 0.551).

**Figure 5:**
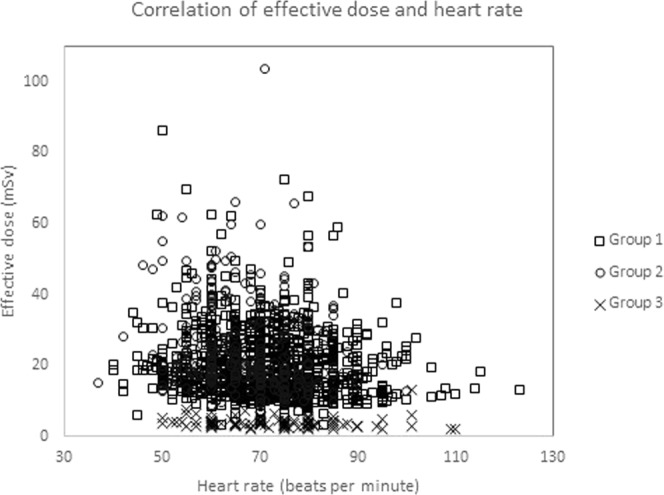
The graph shows the correlation analysis of the estimated effective dose depending on heart rate. Weak negative correlation was shown in all three groups (Group 1, r = −0.132; Group 2, r = −0.158; and, Group 3, r = −0.195).

As illustrated in Fig. 4, the Spearman’s correlation analysis for all three groups showed a significant positive correlation between BMI and estimated effective dose (*p* < 0.001). This showed that the estimated effective dose would increase as the BMI increased. However, Fig. 5 showed an inverse correlation between heart rate and the estimated effective dose.

## Discussion

This study evaluates several factors that influence radiation dose exposure performed using routine CCTA examination protocols at IJN. We obtained compelling evidence that the protocols were exposing patients to considerably higher radiation doses. These findings concurred with the global CTA dose survey published recently, where the DLP value of retrospective ECG-gated from a 2007 survey was higher than the latest survey, which was dominated by prospective ECG-gated scans, by a factor of 7^13^. It can, therefore, be reasonably assumed that our current imaging practices might be causing the high radiation dose exposure rather than existing variables. It should be noted that retrospective ECG-gated CCTA used a lower pitch factor, which was the reason for the high radiation dose exposure on patients.

It is highly recommended for a cardiac imaging centre to apply dose optimisation protocols, but that would be highly dependent on the patients’ heart rate. The main obstacle would be patients with high heart rate because tube current could not be modulated fast enough to catch up with the heartbeat, which would compromise image quality^13,16^. Therefore, to ensure that the ECG-gated tube current modulation was effective in reducing radiation dose exposure, beta-blockers were prescribed to lower the patients’ heart rate to an optimal range^17^.

However, our results showed the mean estimated effective dose was lower in patients with a higher heart rate (>65 bpm). A previous study showed similar results, where the mean effective dose for DSCT was observed to be 11.4 mSv, and it was reduced to 3.8 mSv after applying a tube voltage protocol of 100 kV with 110 ms of ECG pulsing window^18^.

Furthermore, previous studies also reported a decreased dose of radiation received by patients with higher heart rates when applying the recommended ECG-pulsing window technique^19,20^. We altered the ECG-pulsing window width based on heart rate during the examination and, therefore, the differences in estimated radiation dose might be partly explained by the individual ECG-pulsing window widths. Another variable that affected radiation dose exposure was tube voltage, where applying a lower voltage could be expected to reduce the dose^21^. Our study adapted the tube voltage to the patients’ size and weight, and a significant difference were observed in radiation doses between 140 kVp, 120 kVp and 100 kVp in Group 1 and 3 patients (except 160 kVp). Reducing the tube voltage from 120 kVp to 100 kVp could decrease the effective dose by 28%, which was corroborated by earlier findings^22^.

However, tube voltage had to be applied proportionally to BMI because higher penetration energy required to scan patients with more body mass. Reducing the tube voltage might compromise image quality, particularly in obese patients^23,24^. The low voltage would lead to lower photon energy being produced in the scanning tube, resulting in an increase of image noise, artifacts and a decrease of contrast range. Thus, a few studies suggested that low voltage should be used frequently in non-obese or patients with low BMI only^25–27^.

In Group 2, the mean effective dose with a tube voltage of 120 kVp was shown to be higher than 140 kVp. This contradictory result could be due to the change of acquisition protocols, where the technologist might have increased the reference mAs to reduce image noise. This observation was also supported by the results in Table 3, where the scanning time in Group 2 was higher by a factor of 1.3 compared with Group 1 due to the long scan range in post-CABG patients. The scanning range for post-CABG patients started from the arch of the aorta until the heart apex to include the ligation of the coronary artery bypass^3^. As part of the optimization process, the radiation dose from CT scans could be reduced with a few techniques, such as using an iterative reconstruction algorithm, increasing the helical pitch and lowering the tube potential energy^27–29^. Therefore, lowering the tube voltage to reduce effective radiation dose was practical for small- and average-sized patients, but not for those with large habitus^15,30^.

Our study showed a significant positive correlation between BMI and effective dose. Therefore, BMI might be one variable that could contribute to changes in radiation dose. The automatic tube current modulation in the CT scanner would operate according to size and attenuation of body region, and therefore, obese patients would significantly receive higher dose of scanning radiation.

Although an individual with BMI of 30 and above could be assumed to have excess fat mass, the distribution of fat tissues was different in the chest which, in fact, was influenced by the Compton scattering effect^31^. The E/DLP value of 0.026 mSvmGy^−1^cm^−1^ used to calculate the estimated effective dose was obtained based on the International Commission on Radiological Protection (ICRP) report^15^. The value was more accurate for estimating cardiac CT radiation dose compared with chest CT examination^3,29^. The cardiac region had been reported to be more radiosensitive than the chest and the tissue weighting factor was changed significantly from 0.05 to 0.12 for breast as reported in ICRP-103^32^.

In retrospective ECG-gating CCTA examination, it was observed that patients with normal heart rate or below 65 bpm would receive low radiation exposure with high diagnostic image quality produced. But recent development of CT scanners, especially dual-source CT, a high heart rate would no longer be an issue in retrospective CCTA scanning^7^. Therefore, several methods had been suggested to overcome this issue, such as adaptive sequential scanning (prospective ECG-gated), especially when using high-end scanners. In addition, padding window in prospective ECG-gated is a solution for scanning patients with higher heart rate, but this technique might cause interference in temporal resolution.

We aware that our study might have limitations. Firstly, it was a retrospective analysis, where many parameters were not strictly controlled, such as the selection of tube voltage protocols and the recording of patient data. Secondly, it was inevitable that the estimated effective dose values were higher when compared with other studies. The reasons were wholly understood because of the current practice, which caused significant increase in radiation dose as well as other factors. The estimated effective dose was derived from a mathematical formula, which might under- or overestimate the true radiation exposure. Therefore, a validation test should be performed by statistically comparing the doses with the size-specific dose estimation (SSDE). But that was not performed in this study because the CT dose index (CTDI) was not recorded.

In conclusion, a higher heart rate, lower tube voltage and lower BMI might reduce radiation dose significantly in a retrospective ECG-gating protocol using DSCT. It is advantageous to use ECG-pulsing protocol in DSCT for patients with a high heart rate. To perform CCTA safely, patient data must be taken into consideration and a lower tube voltage should be chosen.
